# The cardiac repair benefits of inflammation do not persist: evidence from mast cell implantation

**DOI:** 10.1111/jcmm.12703

**Published:** 2015-10-16

**Authors:** Zhengbo Shao, Mansoreh Nazari, Lily Guo, Shu‐Hong Li, Jie Sun, Shi‐Ming Liu, Hui‐Ping Yuan, Richard D. Weisel, Ren‐Ke Li

**Affiliations:** ^1^ Division of Cardiovascular SurgeryToronto General Research InstituteUniversity Health NetworkTorontoONCanada; ^2^ Division of Cardiac SurgeryDepartment of SurgeryUniversity of TorontoTorontoONCanada; ^3^ Department of OphthalmologySecond Affiliated Hospital of Harbin Medical UniversityHarbinChina; ^4^ Department of CardiologySecond Affiliated Hospital of Guangzhou Medical UniversityGuangzhou Institute of Cardiovascular DiseaseGuangzhouChina

**Keywords:** c‐Kit‐deficient mice, mast cells, myocardial infarction, inflammatory response, cell transplantation, myofibroblasts

## Abstract

Multiple mechanisms contribute to progressive cardiac dysfunction after myocardial infarction (MI) and inflammation is an important mediator. Mast cells (MCs) trigger inflammation after MI by releasing bio‐active factors that contribute to healing. c‐Kit‐deficient (*Kit* ^*W/W‐v*^) mice have dysfunctional MCs and develop severe ventricular dilatation post‐MI. We explored the role of MCs in post‐MI repair. Mouse wild‐type (WT) and *Kit* ^*W/W‐v*^ MCs were obtained from bone marrow (BM). MC effects on fibroblasts were examined *in vitro* by proliferation and gel contraction assays. MCs were implanted into infarcted mouse hearts and their effects were evaluated using molecular, cellular and cardiac functional analyses. In contrast to WT,* Kit* ^*W/W‐v*^ MC transplantation into *Kit* ^*W/W‐v*^ mice did not improve cardiac function or scar size post‐MI. *Kit* ^*W/W‐v*^ MCs induced significantly reduced fibroblast proliferation and contraction compared to WT MCs. MC influence on fibroblast proliferation was Basic fibroblast growth factor (bFGF)‐dependent and MC‐induced fibroblast contractility functioned through transforming growth factor (TGF)‐β. WT MCs transiently rescue cardiac function early post‐MI, but the benefits of BM cell implantation lasted longer. MCs induced increased inflammation compared to the BM‐injected mice, with increased neutrophil infiltration and infarct tumour necrosis factor‐α (TNF‐α) concentration. This augmented inflammation was followed by increased angiogenesis and myofibroblast formation and reduced scar size at early time‐points. Similar to the functional data, these beneficial effects were transient, largely vanishing by day 28. Dysfunctional *Kit* ^*W/W‐v*^ MCs were unable to rescue cardiac function post‐MI. WT MC implantation transiently enhanced angiogenesis and cardiac function. These data suggest that increased inflammation is beneficial to cardiac repair, but these effects are not persistent.

## Introduction

Myocardial infarction (MI) results in dramatic cardiomyocyte death, which in turn activates innate immune mechanisms, initiating an inflammatory response. Inflammation and cytokine elaboration contribute to wound healing and cardiac remodelling after an MI. Mast cells are part of the innate and adaptive immune systems and mainly reside in proximity to vessels. These cells are capable of secreting a wide array of inflammatory and pro‐fibrotic mediators. MC density increases upon MI 1, 2, after which the MCs promptly degranulate, releasing large amounts of bioactive mediators such as histamine and TNF‐α, initiating a cytokine cascade that contributes to the early inflammatory phase of healing 3 This phase is associated with the recruitment and accumulation of leucocytes in the injured area and their release of free radicals and enzymes that clear the dead cells and matrix debris 4. In addition, MC granules contain a wide range of growth, angiogenic and extracellular matrix modulatory factors which enable them to influence matrix remodelling and contribute to granulation and scar formation 5.

Mast cells originate from pluripotent progenitor cells in the BM and mature under the influence of the c‐Kit ligand, stem cell factor (SCF) and the microenvironment of their final destination tissue. Development of MCs is crucially dependent on SCF‐induced Kit dimerization and auto‐phosphorylation. c‐Kit‐deficient (*Kit* ^*W/W‐v*^) mice are heterozygous for the truncated *W* allele (defective in surface expression of Kit) and the point‐mutated *W* ^*v*^ allele (lacking Kit catalytic activity) and as a result suffer from severe MC deficiency 6, 7. Previous studies in our laboratory indicated that *Kit* ^*W/W‐v*^ mice experience impaired myocardial healing with pronounced LV dilatation and expanded scar size after an MI 8, 9. However, replacement of the mutant BM with wild‐type (WT) BM cells completely restored cardiac function and prevented progressive ventricular dilation. These data suggest that inflammation is important for wound healing and cardiac regeneration after MI.

Considering the severe MC deficiency of *Kit* ^*W/W‐v*^ mice and the capability of MCs to moderate the healing process in infarcted regions, we has been suggested that defective wound healing after MI in *Kit* ^*W/W‐v*^ mice may be associated with the lack of cardiac‐resident MCs. Here, we examined the impact of MCs on the infarct healing process following an MI.

## Materials and methods

Detailed procedural descriptions are given in the Data S1.

### Animal procedures and cell preparation

Animals received humane care according to the *Guide for the Care and Use of Laboratory Animals, 8* ^*th*^ *edition* (NIH, revised 2011) and all experimental procedures were approved by the Animal Care Committee of the University Health Network.

Bone marrow cells and MCs were obtained by flushing the marrow cavities of 6‐ to 8‐week‐old *Kit* ^*W/W‐v*^ or WT C57BL/6 mice. MCs were cultured in 5% OPTI‐MEM containing 6% WEHI‐3. After 1 month in culture, MC quality was confirmed by flow cytometry and toluidine blue staining. Cells with toluidine blue‐positive granules and that were >97% positive for c‐Kit and for FcεRI‐α and >90% double‐labelled for both markers were used in experiments (Fig. S1).

To study MC function *in vivo*, female mice underwent ligation of the left anterior descending coronary artery. Cell transplantation (3 × 10^5^ cells) was performed immediately following ligation in three injections across the area subtended by the ligated artery. *Kit* ^*W/W‐v*^ mice were injected with *Kit* ^*W/W‐v*^ MCs or media alone, and WT C57BL/6 mice were injected with WT C57BL/6 MCs or media alone (*n* = 5/group). To compare the effects of MC and BM cell transplantation, C57BL/6 WT mice were implanted with WT C57BL/6 MCs, WT C57BL/6 BM cells or media alone (*n* = 9/group).

### Cardiac function and morphometry

Cardiac function was evaluated by echocardiography and pressure–volume analysis. At 7 and 28 days post implantation, animals were killed and hearts were arrested, fixed, sectioned, and photographed. Scar area was measured by computed planimetry using ImageJ software (National Institutes of Health, Bethesda, Maryland, USA) and expressed as percentage area of the LV free wall.

### *In vitro* assays

Wild‐type C57BL/6 fibroblasts were co‐cultured with WT or *Kit* ^*W/W‐v*^ MCs with or without bFGF or an FGF‐2 neutralizing antibody and cell proliferation was measured by an MTT assay up to 6 days after plating.

Wild‐type C57BL/6 fibroblasts were co‐cultured with WT or *Kit* ^*W/W‐v*^ MCs in collagen gels with or without recombinant TGF‐β or a TGF‐β‐neutralizing antibody and gel contraction was measured 2 days later.

### ELISA and immunohistochemistry

The concentrations of bFGF, TGF‐β and TNF‐α in infarcted and non‐infarcted heart regions were measured by ELISA 3 and 7 days post‐MI and cell transplantation. Isolectin staining identified capillaries and α‐smooth muscle actin (α‐SMA) staining (excluding blood vessel structures) was used to identify myofibroblasts in heart sections on day 3, 7 and 28 post‐MI and cell transplantation. The mobilization of the sub‐type of monocyte/macrophage in the infarct and peri‐infarct areas was identified with immunofluorescence labelling for Ly‐6C and CD11b (Alexa488 antimouse Ly‐6C, eBiosciences and APC conjugated antimouse CD11b; BD Biosciences, San Jose, CA, USA). Total leucocyte infiltration was assessed with CD45 (BD Biosciences) immunolabelling.

### Flow cytometry

Mast cells were labelled with PE‐conjugated antibodies against c‐Kit and FcεRI‐α. PE‐conjugated IgEhb served as the isotype control.

Hearts were separated into infarct and non‐infarct segments prior to digestion. A total of 10^6^ cells were stained with FITC‐conjugated rat antimouse neutrophil and rat antimouse F4/80 antibodies. Isotype‐identical antibodies served as controls. Cells were analysed by flow cytometry.

### Statistics

Data are presented as mean ± S.D. Comparisons among three or more groups were performed with anova, with differences specified by Tukey or Bonferroni *post hoc* tests. A value of *P* < 0.05 was considered statistically significant. All statistical analyses were performed with GraphPad Prism 5.0. (La Jolla, CA, USA).

## Results

### Implantation of MCs from Kit^W/W‐v^ mice could not rescue impaired cardiac function of Kit^W/W‐v^ mice after MI

**Figure 1:**
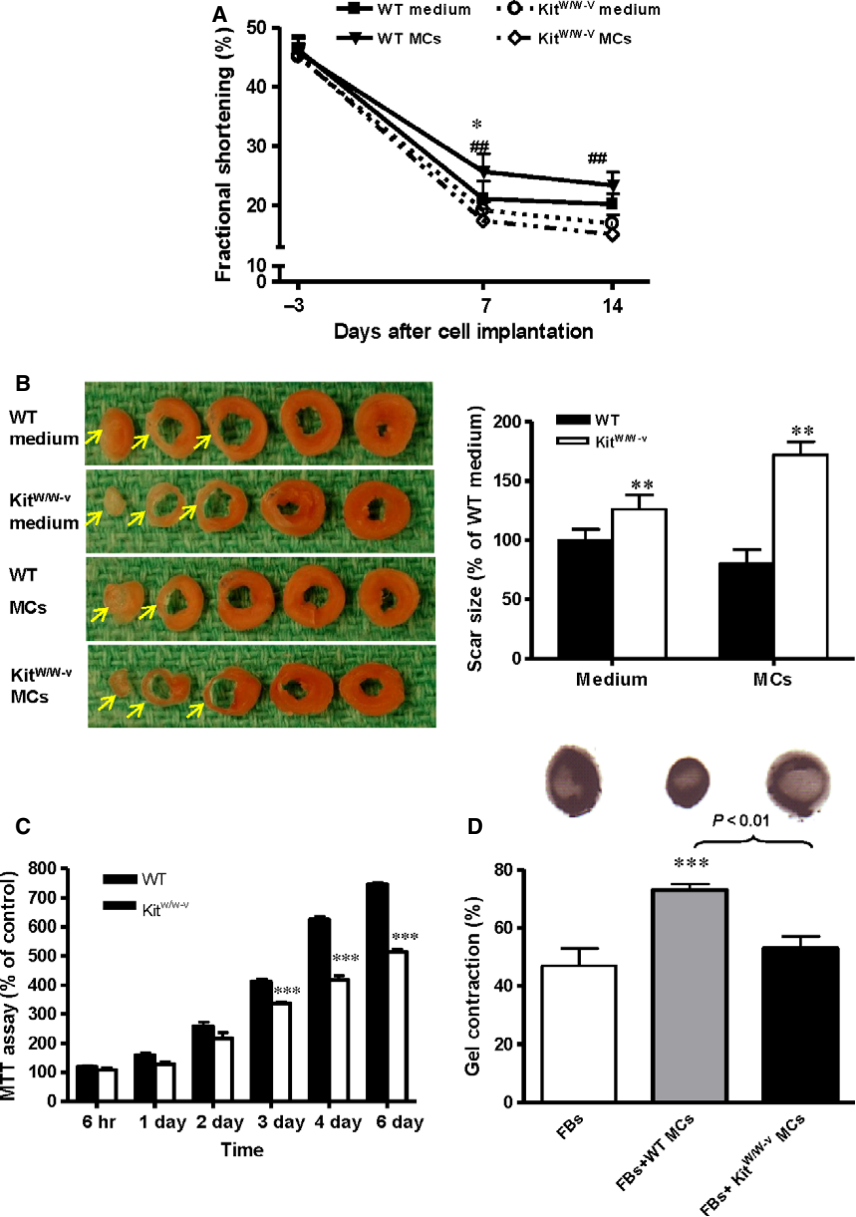
Impaired mast cells (MCs) are associated with deterioration of cardiac function in *Kit* ^*W/W‐v*^ mice after ischaemic injury. (**A**) Wild‐type (WT) and *Kit* ^*W/W‐v*^ mice underwent myocardial infarction and received isogenic (WT and *Kit* ^*W/W‐v*^) MCs or medium alone. Per cent fractional shortening was evaluated by echocardiography before (day −3) and after (days 7 and 14) infarction (*n* = 5, *P < 0.05 for WT MCs *versus* WT medium‐injected; ##*P* < 0.01 for *Kit* ^*W/W‐v*^ MCs *versus* WT MCs). (**B**) Whole heart serial sections demonstrated increased scar size 14 days after implantation with isogenic MCs in *Kit* ^*W/W‐v*^ mice compared to WT recipients of WT MCs (*n* = 5, **P < 0.01 *versus* WT). (**C**) An MTT assay demonstrated that co‐culture with WT MCs increased WT fibroblast (FB) proliferation compared to co‐culture with *Kit* ^*W/W‐v*^ MCs (*n* = 3, ***P < 0.001 *versus* WT). (**D**) A gel contraction assay demonstrated co‐culture with WT MCs increased the contractile function of WT FBs, while co‐culture with *Kit* ^*W/W‐v*^ MCs did not (*n* = 3, ***P < 0.001 *versus* FBs). Gel contraction is expressed as the per cent shrinkage compared with a control gel without cells.

Systolic cardiac function was evaluated by echocardiography in WT and *Kit* ^*W/W‐v*^ mice receiving MCs produced from the BM of isogenic mice or medium alone. Before MI, there was no difference in fractional shortening, an index of systolic function, between the groups. At day 7 post‐MI, fractional shortening was significantly better in the WT mice that received WT MCs compared to the WT mice receiving medium alone (Fig. 1A, *P* < 0.05). This functional improvement was transient, and by day 14 non‐significant difference was observed. However, for the *Kit* ^*W/W‐v*^ mice, implantation of *Kit* ^*W/W‐v*^‐derived MCs did not improve fractional shortening at any time‐point compared to medium alone. The difference between WT and *Kit* ^*W/W‐v*^ mice was not evident when medium was injected into the heart, but WT mice had significantly better fractional shortening than *Kit* ^*W/W‐v*^ mice following isogenic MC implantation (*P* < 0.01 at days 7 and 14).

Heart morphometry evaluation of WT and *Kit* ^*W/W‐v*^ mice demonstrated that infarct scar size was significantly greater in the *Kit* ^*W/W‐v*^ mice compared to the WT mice for both MC implantation and medium alone groups (Fig. 1B, *P* < 0.01).

Co‐culture with MCs has been shown to promote fibroblast contraction and proliferation 10, 11. To evaluate the efficiency of MCs prepared from *Kit* ^*W/W‐v*^ mice, an MTT assay and a collagen gel contraction assay were employed to assess the proliferative and contractive effects of these cells on fibroblasts. Wild‐type MCs imposed stronger proliferative effects on WT fibroblasts compared to MCs derived from *Kit* ^*W/W‐v*^ mice (Fig. 1C, *P* < 0.001 at 3, 4 and 6 days after plating). Similarly, a three‐dimensional collagen gel contraction assay confirmed the contractile force generated by fibroblasts co‐cultured with WT MCs was significantly stronger than WT fibroblasts alone or WT fibroblasts co‐cultured with *Kit* ^*W/W‐v*^ MCs (Fig. 1D, *P* < 0.01). No significant difference in contraction was noticed between fibroblasts cultured alone and those cultured with *Kit* ^*W/W‐v*^ MCs. These results suggest MCs derived from *Kit* ^*W/W‐v*^ mice are less efficient than WT MCs at promoting contraction and proliferation in neighbouring fibroblasts.

### Functional MCs increased cardiac fibroblast proliferation through bFGF

**Figure 2:**
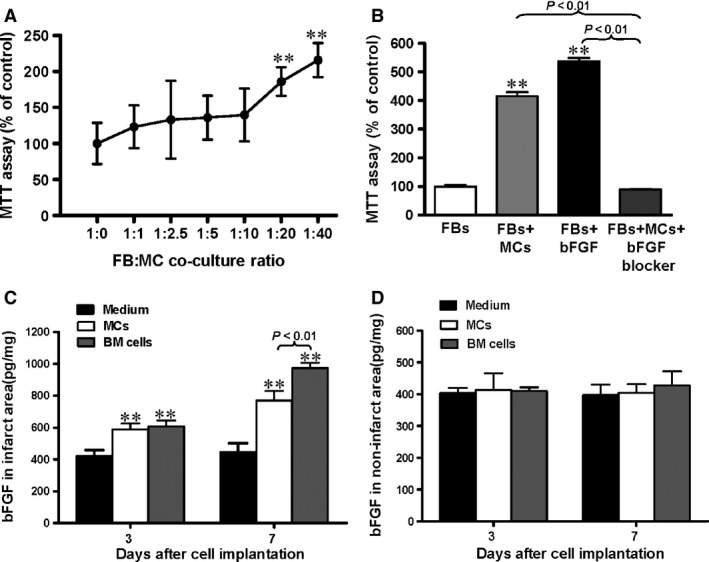
Functional mast cells (MCs) increased cardiac fibroblast (FB) proliferation through bFGF. (**A**) Co‐culture with WT MCs induced dose‐dependent increased proliferation of cardiac WT FBs by an MTT assay. Data are expressed as % proliferation compared to FBs alone (*n* = 8, *P* < 0.01 *versus* 1:0 FBs:MCs). (**B**) After 48 hrs co‐culture, MCs induced proliferation of FBs to a similar extent as stimulation with bFGF, and the effect was prevented by an FGF‐2 neutralizing antibody (*n* = 3, **P < 0.01 *versus* FBs alone). bFGF expression was increased by MC or bone marrow (BM) cell implantation in the infarcted (**C**) but not the non‐infarcted (**D**) myocardium at 3 and 7 days after implantation as assessed by ELISA (*n* = 6, **P < 0.01 *versus* medium‐injected control).

Next, to evaluate the mechanism behind the effects of functional MCs on cardiac fibroblast proliferation, WT MCs derived from C57/B6 mice were co‐cultured with WT C57/B6 fibroblasts. MTT results showed that the proliferative effect of MCs on fibroblasts was dose‐dependent, with increasing proportions of MCs corresponding with higher fibroblast proliferation (Fig. 2A, *P* < 0.01 for 1:20 and 1:40 fibroblasts:MCs *versus* fibroblasts alone). These results are consistent with a pro‐proliferative mediator being released from MCs in co‐culture in an MC concentration‐dependent manner. bFGF is a pro‐proliferative mediator which is released from MCs 12. We found that the proliferative effect of MCs on fibroblasts is bFGF‐dependent. Similar fibroblast proliferation was measured in the presence of MCs or bFGF (*P* < 0.01 compared to fibroblasts alone), and an FGF‐2 neutralizing antibody completely abolishes the proliferative effects of MCs (Fig. 2B, *P* < 0.01). These results suggest the proliferative effects of MCs on fibroblasts are mediated through bFGF.

To assay whether MC transplantation induced increased local bFGF concentrations *in vivo*, ELISA was used to measure expression of bFGF in infarct and non‐infarct zones after cell implantation at day 3 and 7 post‐MI (Fig. 2C and D). There was a significant increase in bFGF expression in the infarct region of the mice implanted with MCs and BM cells at both time‐points compared to the medium‐injected group (Fig. 2C, *P* < 0.01). However, bFGF expression was significantly higher in animals injected with BM cells than MCs at day 7 (*P* < 0.01). No significant difference in bFGF level was seen between different groups in the non‐infarcted region (Fig. 2D).

### Functional MCs induce cardiac fibroblast‐to‐myofibroblast conversion through TGF‐β

**Figure 3:**
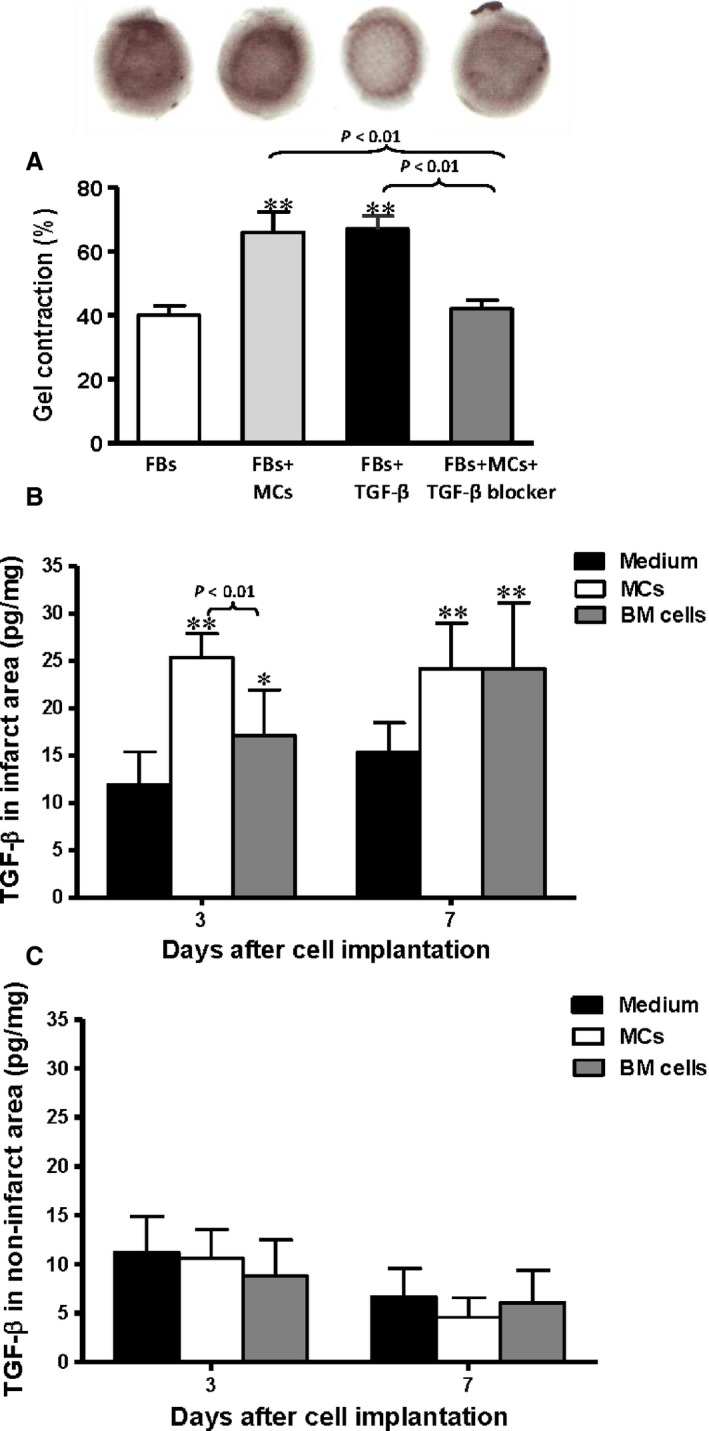
Functional mast cells (MCs) induced cardiac fibroblast‐ (FB‐) to‐myofibroblast conversion through TGF‐β. (**A**) Wild‐type MCs induced the contraction of WT FBs in a collagen gel contraction assay to a similar extent as exogenous TGF‐β, and the effect was prevented by a TGF‐β neutralizing antibody. Gel contraction is expressed as the per cent shrinkage compared with a control gel without cells (*n* = 3, *P < 0.01 *versus* FBs alone). TGF‐β expression was increased in (**B**) infarcted but not in (**C**) non‐infarcted regions of the myocardium after MC and bone marrow (BM) cell transplantation 3 and 7 days after myocardial infarction and cell implantation by ELISA (*n* = 6, *P < 0.05 and **P < 0.01 *versus* medium‐injected).

The conversion of fibroblasts to contractile myofibroblasts is controlled largely through TGF‐β, which is also secreted from MCs 13. Collagen gels seeded with cardiac fibroblasts will contract in proportion to the number of the fibroblasts that have differentiated to myofibroblasts. A three‐dimensional collagen gel contraction assay was used to determine the paracrine influence of MCs on fibroblast‐to‐myofibroblast differentiation by measuring the contractile force of the cells (Fig. 3A). Co‐culture of fibroblasts with WT MCs or exogenous TGF‐β resulted in significant gel contraction compared to the fibroblast‐only group (*P* < 0.01). The effect of MCs was approximately equal to that of exogenous TGF‐β. The addition of a TGF‐β neutralizing antibody to the fibroblast/MC co‐culture abolished the pro‐contraction effect of MCs (*P* < 0.01). These results suggest the pro‐contractile effects of MCs on fibroblasts are mediated through TGF‐β.

We has been suggested MC‐derived TGF‐β may play a role in the transient improvement seen after MC transplantation post‐MI. The amount of TGF‐β protein in the infarcted and non‐infarcted areas of the myocardium after cell implantation was measured using ELISA (Fig. 3B and C). Mast cell implantation significantly increased TGF‐β expression in the infarct region at day 3 and 7 post‐MI compared to the medium‐injected control group (*P* < 0.01). Similarly, BM cell implantation also augmented the level of TGF‐β significantly in the infarct region compared to the medium‐injected control group (*P* < 0.05 at day 3, *P* < 0.01 at day 7). However, the concentration of TGF‐β was significantly greater in the MCs group compared to the BM cell group at day 3 (Fig. 3B, *P* < 0.01). In the non‐infarcted area, the amount of TGF‐β remained low in all groups, and no significant differences were seen (Fig. 3C).

### MC implantation transiently improves cardiac function in C57BL/6 mice

**Figure 4:**
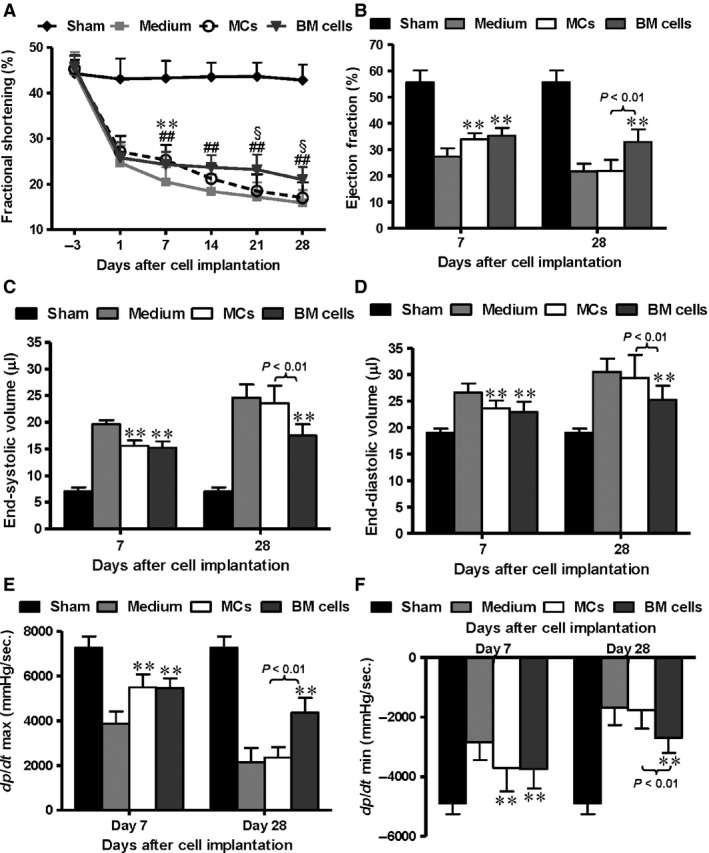
Mast cell (MC) transplantation transiently improved mouse cardiac function post‐myocardial infarction (MI). C57BL/6 mice underwent coronary ligation to induce MI, and wild‐type MCs, bone marrow (BM) cells or medium alone were injected into the myocardium. Sham‐operated mice underwent thoracotomy but no ligation. (**A**) All ligated groups exhibited a drastic decrease in fractional shortening at day 1 after MI assayed by echocardiography (*n* = 9). The MC and BM recipient groups both showed early improvement at day 7. These improvements were lost in the MC group but persisted up to day 28 in the BM group (**P < 0.01 for MCs *versus* medium alone; ##*P* < 0.01 for BM cells *versus* medium alone; §*P* < 0.05 for MCs *versus* BM). Pressure–volume analysis at day 7 and 28 after cell implantation was used to evaluate ejection fraction (**B**), end‐systolic and end‐diastolic volumes (**C** and **D**), and dP/dt max and dP/dt min (**E** and **F**,* n* = 9, **P < 0.01 *versus* medium alone). In all cases, the benefits of MC transplantation were apparent at day 7, but absent by day 28.

Serial echocardiography demonstrated that functional MC and BM cell implantation prevented a further decline in fractional shortening after the abrupt initial decline observed during the first days following MI (Fig. 4A). Fractional shortening progressively decreased in the medium‐injected control group after ligation, but it was transiently improved at day 7 by MC implantation (*P* < 0.01). The BM cell group exhibited better fractional shortening than the medium control group at 7 days following cell implantation, and this improvement was sustained (*P* < 0.01 for days 7–28).

On day 7 and 28 after cell implantation, the ejection fraction of mice implanted with BM cells was significantly better compared to mice receiving medium alone (Fig. 4B, *P* < 0.01). MC transplantation improved ejection fraction early, on day 7 (*P* < 0.01). End‐systolic and end‐diastolic volumes showed a similar temporal pattern, with a significant improvement in the MC group on day 7 and an improvement in the BM cell group on both days 7 and 28 compared with the medium control (Fig. 4C and D, *P* < 0.01). Similar observations were made for dP/dt max and dP/dt min (Fig. 4E and F, *P* < 0.01). In the BM cell group, the improvement in systolic and diastolic function persisted over 28 days while MC implantation only transiently increased these functional parameters.

### MC implantation is associated with augmentation of the inflammatory response

Mast cells function to increase inflammation post injury and we have been that MC implantation would increase local inflammation post‐MI. Flow cytometry was performed to evaluate the accumulation of neutrophils and macrophages in the infarct and non‐infarct areas of the myocardium at days 1, 3, and 7 post‐MI. The expression of TNF‐α, a pleiotropic inflammatory cytokine, was assessed by ELISA. These three parameters were evaluated as markers of inflammation.

**Figure 5:**
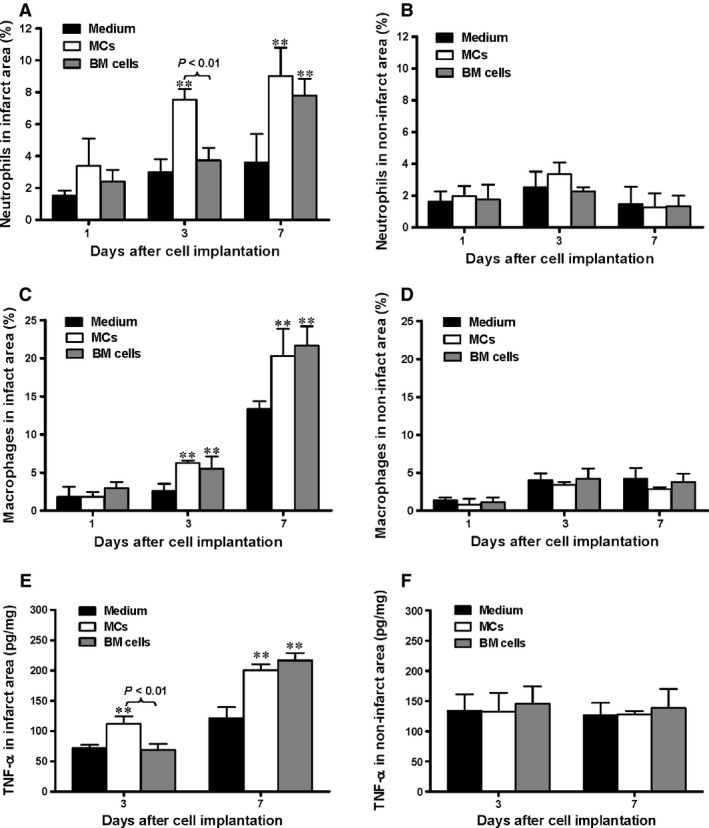
Functional mast cell (MC) implantation is associated with augmentation of the inflammatory response. Infiltration of neutrophils into the (**A**) infarct region was increased at day 3 post infarction for the MC‐implanted group and at day 7 for both the MC‐ and bone marrow (BM) cell‐implanted groups while (**B**) no differences were seen in the non‐infarct regions with or without cell transplantation, evaluated by flow cytometry (*n* = 3, **P < 0.01 *versus* medium alone). Infiltration of macrophages into the (**C**) infarct region was increased in cell‐implanted mice at both time‐points while the (**D**) non‐infarct region saw no increase, evaluated by flow cytometry against F4/80 (*n* = 3, **P < 0.01 *versus* medium alone). TNF‐α concentration in the (**E**) infarct increases by 3 days after MC implantation and by 7 days after BM cell implantation while (**F**) no such increase was seen in the non‐infarcted regions, assessed by ELISA (*n* = 6, **P < 0.01 *versus* medium alone).

One day after MI, there were few neutrophils in the infarct area of the myocardium. However, by day 3, the percentage of neutrophils increased significantly in the MC‐transplanted group compared to the BM cell and medium control groups (Fig. 5A, *P* < 0.01). At day 7 post‐MI, neutrophils in the infarct area increased to a similar level in the MC and BM groups, and both were higher than in the medium‐injected group (*P* < 0.01). The number of neutrophils in the non‐infarct area remained low in all groups at all time‐points (Fig. 5B).

The percentage of macrophages in the infarct area was low initially, but on days 3 and 7, macrophage infiltration was similar in both the MC and BM groups and was significantly higher in these groups compared to the medium‐injected control group (Fig. 5C, *P* < 0.01). In the non‐infarct area, the percentage of macrophages remained low in all groups (Fig. 5D).

The expression of TNF‐α in the infarct area 3 days after MI was significantly higher in the MC group compared to both the medium‐injected control and BM groups (Fig. 5E, *P* < 0.01). Tumour necrosis factor‐α concentration increased in both the MC and BM cell groups at day 28 and was significantly higher than in the control group (Fig. 5E, *P* < 0.01). The concentration of TNF‐α in the non‐infarcted myocardium was not significantly changed (Fig. 5F).

**Figure 6:**
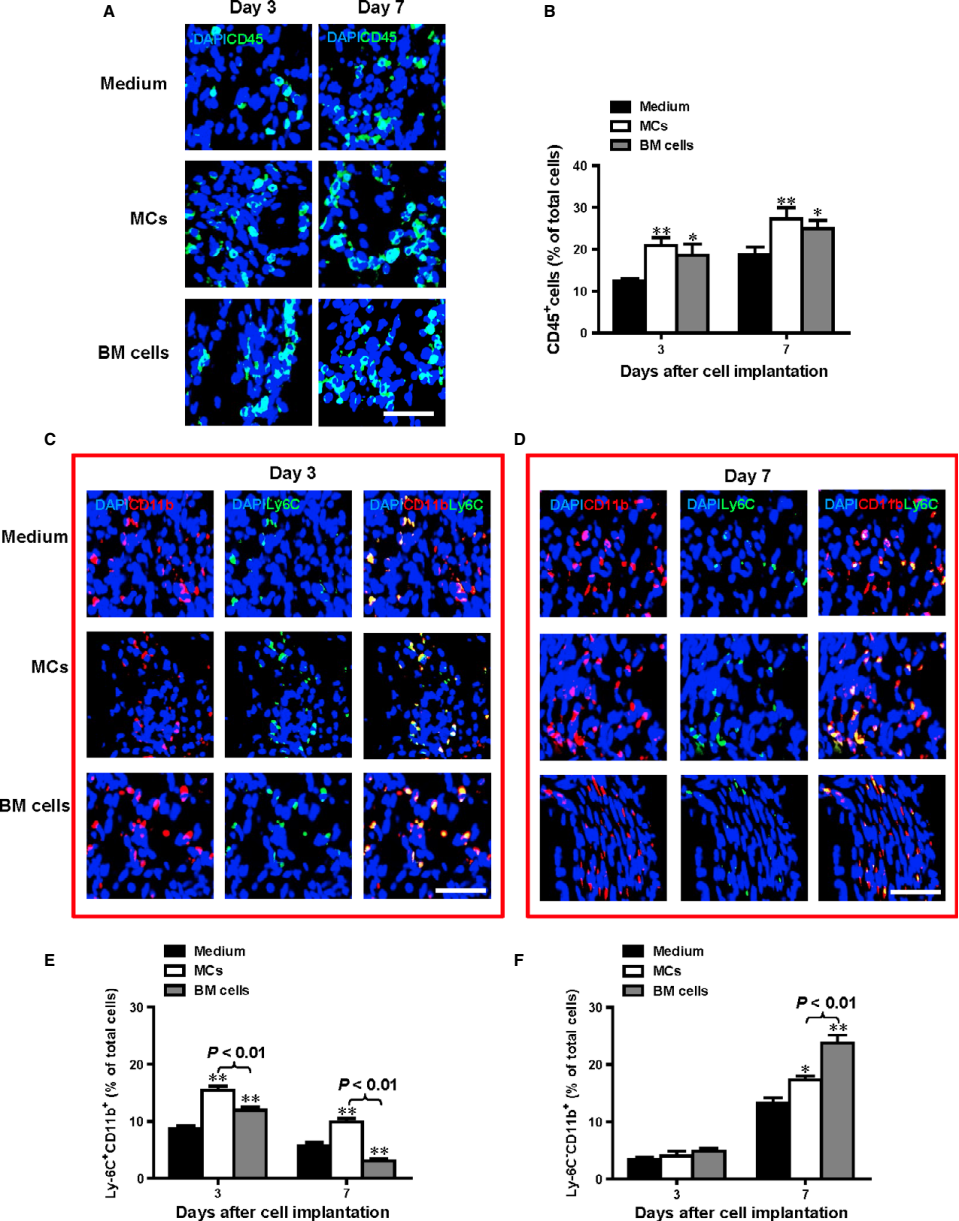
The healing myocardium sequentially mobilizes two monocyte subsets. Infiltration of leucocyte into the infarct region (CD45^+^ cells **A** and **B**) was increased at day 3 and 7 post infarction for both the MC‐ and bone marrow (BM) cell‐implanted groups while no differences were seen in the medium alone group without cell transplantation, evaluated by immunofluorescent labelling (*n* = 3, *P < 0.05, **P < 0.01 *versus* medium alone). Infiltration of Ly‐6C^hi^/CD11b^+^ monocytes peaked at 3 days post‐MI (**C** and **E**,* n* = 3, **P < 0.01 *versus* medium alone) whereas the Ly‐6C^lo^/CD11b^+^ peaked at 7 days post‐MI (**D** and **F**,* n* = 3, *P < 0.05, **P < 0.01 *versus* medium alone) in both the MC and BM groups and was significantly higher in these groups compared to the medium‐injected control group. However, there was more mobilization of Ly‐6C^hi^/CD11b^+^ monocytes in the MC group compared with the BM group at 3 and 7 days post‐MI (**E**,* n* = 3, **P < 0.01). On the other hand, there was more mobilization of Ly‐6C^lo^/CD11b^+^ monocytes in the BM group compared with the MC group at 7 days post‐MI (**F**,* n* = 3, **P < 0.01); scale bar = 50 μm

To further elucidate the mobilization of the sub‐type of monocyte/macrophage in the infarct and peri‐infarct areas (in addition to non‐infarcted areas) after cell transplantation, immunofluorescence labelling for subsets of the monocyte (Ly‐6C and CD11b) were performed. Furthermore, total leucocyte infiltration was assessed with CD45 immunolabelling in the infarct and peri‐infarct areas (in addition to non‐infarcted areas) after cell transplantation. As showed in Figure 6, we found that the Ly‐6C^hi^/CD11b^+^ monocytes peaked at 3 days post‐MI (Fig. 6C and E, **P < 0.01) whereas the Ly‐6C^lo^/CD11b^+^ peaked at 7 days post‐MI (Fig. 6D and F, *P < 0.05) in both the MC and BM groups and was significantly higher in these groups compared to the medium‐injected control group. However, there was more mobilization of Ly‐6C^hi^/CD11b^+^ monocytes in the MC group compared with the BM group at 3 days post‐MI (Fig. 6E, **P < 0.01) though the total number of leucocyte infiltration (CD45^+^ cells, Fig. 6A and B, *P < 0.05) are about the same for the two groups. On the other hand, there was more mobilization of Ly‐6C^lo^/CD11b^+^ monocytes in the BM group compared with the MC group at 7 days post‐MI (Fig. 6F, **P < 0.01). This results were in agreement with previous finding reported by Nahrendorf *et al*. 14 that the healing myocardium sequentially mobilizes two monocyte subsets with divergent and complementary functions whereas the Ly‐6C hi monocytes dominate early (phase I) with phagocytic, proteolytic and inflammatory functions and Ly‐6C lo monocytes dominate later (phase II) with myofibroblast accumulation, angiogenesis and deposition of collagen.

### Enhanced angiogenesis and increased myofibroblast production are associated with improved cardiac function after MC implantation

**Figure 7:**
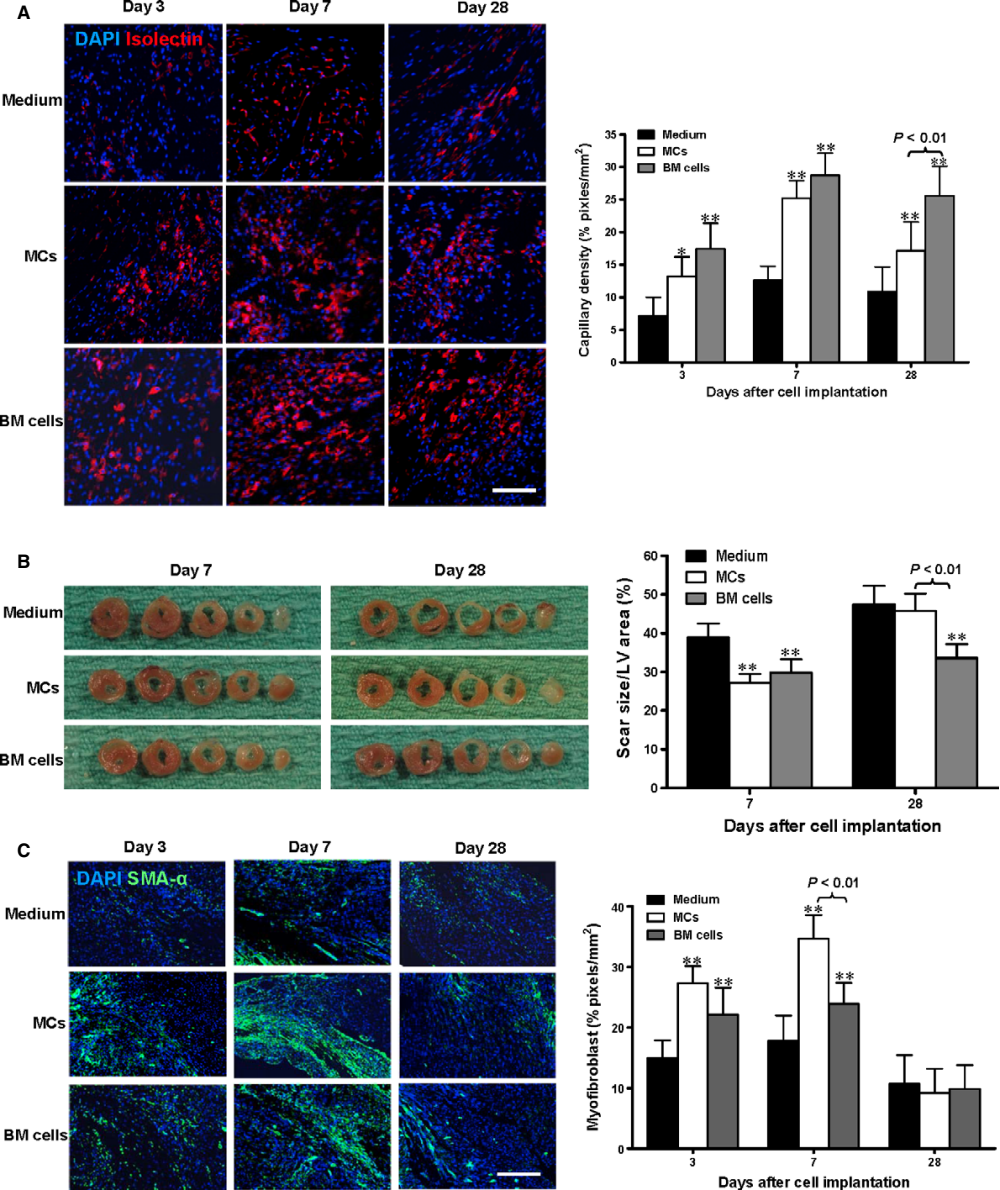
Enhanced angiogenesis is the predominant contributor to improved cardiac function after cell implantation. (**A**) Capillary density in the border zone was increased at day 3, 7 and 28 after wild‐type mast cell (MC) or bone marrow (BM) cell implantation, quantified by isolectin staining (*n* = 5/group,*P < 0.05 and **P < 0.01 *versus* medium‐injected control, scale bar = 100 μm). (**B**) Whole heart serial sections at day 7 and 28 post‐cell implantation illustrate ventricular dilation in MC and medium injected controls at the later time‐point. Infarct scar area was quantified as a percentage of left ventricular (LV) area (*n* = 6/group, **P < 0.01 *versus* medium‐injected control). (**C**) α‐SMA staining for myofibroblast accumulation in the border zone post implantation shows increased myofibroblast accumulation in the MC injected hearts 7 days post transplantation compared to their BM‐injected counterparts. Quantification of the α‐SMA ^+^ area excluded blood vessel structures (*n* = 5/group, **P < 0.01 *versus* medium‐injected control, scale bar = 100 μm).

Isolectin staining of myocardial tissue sections was used to assess the angiogenic effect of cell implantation after MI and demonstrated more blood vessels in the border zone of the MC‐ and BM‐implanted groups compared to the medium‐injected control group (Fig. 7A, *P* < 0.05 for MC‐implanted animals at day 3, *P* < 0.01 for MC‐implanted at day 7 and 28 and BM‐implanted at all time‐points). The angiogenic effect of MC and BM implantation peaked at day 7, and the effect lasted until at least day 28. The BM group had higher capillary density at day 28 than the MC group (*P* < 0.01).

Heart morphometry was assessed at day 7 and 28 after cell implantation (Fig. 7B). In mice receiving MCs, infarct scar size was significantly reduced only at day 7 compared to the medium‐injected control group (*P* < 0.01). In mice receiving BM cells, significant reductions in scar size were detected at day 7 and 28 compared to the mice receiving medium alone (*P* < 0.01). Therefore, the BM cell group showed a relative stabilization in scar size post‐MI, while the MC group showed infarct expansion after day 7 (*P* < 0.01).

Accumulation of myofibroblasts in the injured area after MI was assessed by immunohistochemical staining for α‐SMA at days 3, 7 and 28 following cell implantation (Fig. 7C). Quantification of this staining showed significantly increased α‐SMA expression in both the MC and BM cell groups compared to medium‐injected controls at day 3 and 7 (*P* < 0.01), but the MC group accumulated more myofibroblasts than the BM cell group at day 7 (*P* < 0.01). At day 28, α‐SMA was reduced and there were no differences among the groups.

## Discussion

The c‐Kit receptor is expressed by various types of cells, including endothelial progenitor cells, hematopoietic and resident cardiac stem cells, and MCs 15. *Kit* ^*W/W‐v*^ mice have decreased c‐Kit expression and signal transduction, which is essential to development, survival and migration of MCs, making *Kit* ^*W/W‐v*^ animals MC‐deficient 16, 17. In our previous studies 9, we used *Kit* ^*W/W‐v*^ c‐kit mutant mice and their WT littermates to assess the importance of c‐kit function in cardiac remodelling after coronary ligation. We found that mutant *Kit* ^*W/W‐v*^ mice had decreased mobilization of the cells to the heart, which in turn prevented angiogenesis, diminished myofibroblast‐rich repair tissue formation and led to precipitous cardiac failure and death. Replacement of the mutant BM with WT BM cells completely restored cardiac function and prevented excessive ventricular dilation. Furthermore, BM rescue increased myocardial VEGF level and increased the number of blood vessels in the *Kit* ^*W/W‐v*^ border zone 7 days after MI. Thus, reconstitution of *Kit* ^*W/W‐v*^ BM with WT BM rescued the *Kit* ^*W/W‐v*^ cardiomyopathic phenotype at the biochemical, histological, morphometric and functional levels. In the subsequent study, we found that BM cell transplantaiton with healthy c‐kit cells restored the cardiac myofibroblast response, reduced scar length and prevented cardiac dilatation and dysfunction 14 days after the infarct 8. Mast cell deficiency is not the only deficit in *Kit* ^*W/W‐v*^ mice, but because of the contribution of MCs to cardiac repair, we have been suggested that the inefficient wound healing in these animals might stem from dysfunctional MCs. Mast cells are actively involved in inflammation after MI, which is important for cardiac repair and regeneration, and the effects of MCs on these processes have not been directly investigated. We therefore evaluated the contribution of inflammation to cardiac function restoration by implanting *Kit* ^*W/W‐v*^ MCs into the myocardium of *Kit* ^*W/W‐v*^ mice after MI.

Implantation of *Kit* ^*W/W‐v*^ MCs into *Kit* ^*W/W‐v*^ mice after MI did not stimulate the early improvements in scar remodelling and ventricular function seen in WT mice receiving WT MCs. Fractional shortening was not rescued in *Kit* ^*W/W‐v*^ mice after cell therapy and scar area was increased compared to WT controls. On the other hand, WT MCs implanted into WT mice resulted in transient functional benefits, with systolic function improved at day 7 compared to medium‐injected WT mice. Further investigation indicated that functional MCs from WT mice stimulate WT fibroblasts to proliferate more and generate larger contractile force, while inoperative MCs from *Kit* ^*W/W‐v*^ mice did not promote robust proliferative or contractile effects. Reduced fibroblast proliferation could be associated with the smaller number of accumulated myofibroblasts in the injured area of *Kit* ^*W/W‐v*^ mice that we showed in our previous work 8, and this decrease could contribute to poor contractile function, resulting in greater ventricular dilatation and dysfunction in these mice post‐MI. Indeed, dysfuntional MCs, along with functional deficit in other c‐Kit‐expressing cells in *Kit* ^*W/W‐v*^ mice could have caused the inability of MC therapy to rescue cardiac healing following MI.

Mast cells play an important role in post‐infarction healing by augmenting inflammation and tissue repair through release of proteolytic enzymes, cytokines and growth factors 18. In our study, implantation of functional WT MCs enhanced bFGF concentration only in the infarct area post‐MI. We also showed that the proliferative effects of MCs on fibroblasts were dose‐ and bFGF‐dependent. bFGF is a potent fibroblast proliferative 19 and angiogenic factor 20 produced by MCs, macrophages, and other cells with mesodermal or neuroectodermal origin 12, 20. Mast cells have been shown to influence bFGF concentration in infarct regions by direct secretion or through discharging degrading enzymes which can release bFGF from connective tissue stores 20. Increased concentrations of bFGF may contribute to beneficial myofibroblastic and angiogenic responses following MC implantation.

Mast cells influence both the inflammatory and proliferative phases of wound healing by modulating both cellular and cytokine responses. Recipients of functional MCs had a greater inflammatory response, with increased neutrophil infiltration and TNF‐α expression 3 days after cell implantation compared with BM cell recipients. Neutrophils produce and release VEGF, macrophage inflammatory protein‐1α, and macrophage inflammatory protein‐2 21. The recruitment of more neutrophils may have augmented the angiogenesis observed after cell implantation.

Macrophage infiltration was equally enhanced with functional WT MC and BM cell implantation at day 3 post‐MI, with a trend to greater infiltration in both groups at day 7. Augmentation of macrophage infiltration (with macrophage colony‐stimulating factor) after MI accelerates wound repair and improves ventricular function 22, 23. Macrophages secrete VEGF and other growth factors following engulfment of apoptotic cells, augmenting the angiogenic effect of cell therapy 24. Implantation of WT MCs was associated with greater TGF‐β and TNF‐α in the infarct region. Increased TGF‐β may have stimulated greater myofibroblast accumulation. TNF‐α can be beneficial or detrimental following infarction. Cardiac‐specific expression of TNF‐α can result in increased apoptosis and ventricular dilatation, ultimately leading to heart failure and death 25. However, previous study in our laboratorydemonstrated that TNF‐α also helps attract stem cells to the infarct region 26 and the transient regeneration associated with MC implantation could stem in part from this early increase in TNF‐α.

Inflammation is essential during the onset of the healing process, but sustained inflammation can be associated with activation of cell death pathways. Mast cell implantation augmented the inflammatory response with a dramatic release of TNF‐α, which may contribute to cardiomyocyte apoptosis and limit the beneficial effects of MC implantation. On the other hand, the amplified inflammatory response following MC transplantation was associated with increased angiogenesis and myofibroblastic response, which may result in improved cardiac function.

Mast cells increased the number of myofibroblasts (α‐SMA^+^ non‐vascular cells) in the infarct region early in the post‐MI period (days 3 and 7), but not later at day 28. After an MI, relatively quiescent fibroblasts convert to myofibroblasts, which express contractile proteins such as α‐SMA and produce extracellular matrix components like collagen 27. Myofibroblasts can contract to induce wound closure and contribute to the structural integrity of the healing scar 28, 29, 30. Conversion of fibroblasts to myofibroblasts occurs under the influence of TGF‐β, which is released from macrophages, MCs and necrotic myocytes 18, 27, 30, 31, 32, 33. The increased number of myofibroblasts following MC implantation may have contributed to the improved function and smaller scar size seen early on.

Detailed analysis of the mobilization of the sub‐type of monocyte/macrophage in the infarct and peri‐infarct areas (in addition to non‐infarcted areas) after cell transplantation was done with immunofluorescence labelling for subsets of the monocyte (Ly‐6C and CD11b). Furthermore, total leucocyte infiltration was assessed with CD45 immunolabelling in the infarct and peri‐infarct areas (in addition to non‐infarcted areas) after cell transplantation. Consistent with previous work demonstrated by Nahrendorf *et al*., we found that that the healing myocardium sequentially mobilizes two monocyte subsets with Ly‐6C^hi^ monocytes peaked at early phased (day 3 post‐MI) and Ly‐6C^lo^ peaked at later phase (day 7 post‐MI) of MI. However, there was more mobilization of Ly‐6C^hi^/CD11b^+^ monocytes in the MC group compared with the BM group at 3 days post‐MI though the total number of leucocyte infiltration (CD45^+^ cells) were about the same for the two groups. On the other hand, there was more mobilization of Ly‐6C^lo^/CD11b^+^ monocytes in the BM group compared with the MC group at 7 days post‐MI. Similar to the mobilization of the Ly‐6C^hi^/CD11b^+^ monocytes, the expression of inflammatory mediator, TNF‐α in the infarct area 3 days after MI was significantly higher in the MC group compared to both the medium‐injected control and BM groups. On the other hand, the BM group had higher capillary density at all time‐points than the MC group. These data suggested that the healing myocardium sequentially mobilizes two monocyte subsets with Ly‐6C^hi^ monocytes peaked at early phased (day 3 post‐MI) and Ly‐6C^lo^ peaked at later phase (day 7 post‐MI) of MI. Ly‐6C^hi^ monocytes mostly exhibit inflammatory functions, whereas Ly‐6C^lo^ monocytes promote healing *via* myofibroblast accumulation and angiogenesis.

Overall, our study suggests that MC dysfunction in *Kit* ^*W/W‐v*^ mice contributes to their reduced healing after MI. The beneficial infarct healing effects of WT MCs function through augmentation of the early inflammatory, angiogenic and myofibroblastic responses, while the magnitude of inflammation limits the beneficial effects of MCs post‐MI. These results point to the dual beneficial and detrimental roles of inflammation in the healing process.

## Conflicts of interest

The authors confirm that there are no conflicts of interest.

## Author contribution

MN, S‐ML, H‐PY, RDW and R‐KL designed the experiments, ZS, MN, LG, S‐HL and JS performed the experiments, S‐ML and H‐PY contributed knowledge and ZS, MN, RDW and R‐KL prepared the manuscript.

## Supporting information


**Figure S1** Identification and characterization of mast cells (MCs).

**Data S1** Supplemental methods.
