# Supplementary figures and images for: The cardiac repair benefits of inflammation do not persist: evidence from mast cell implantation
Source: J Cell Mol Med. 2015 Oct 16;19(12):2751–62. doi: 10.1111/jcmm.12703 (PMC4687709; doi:10.1111/jcmm.12703)

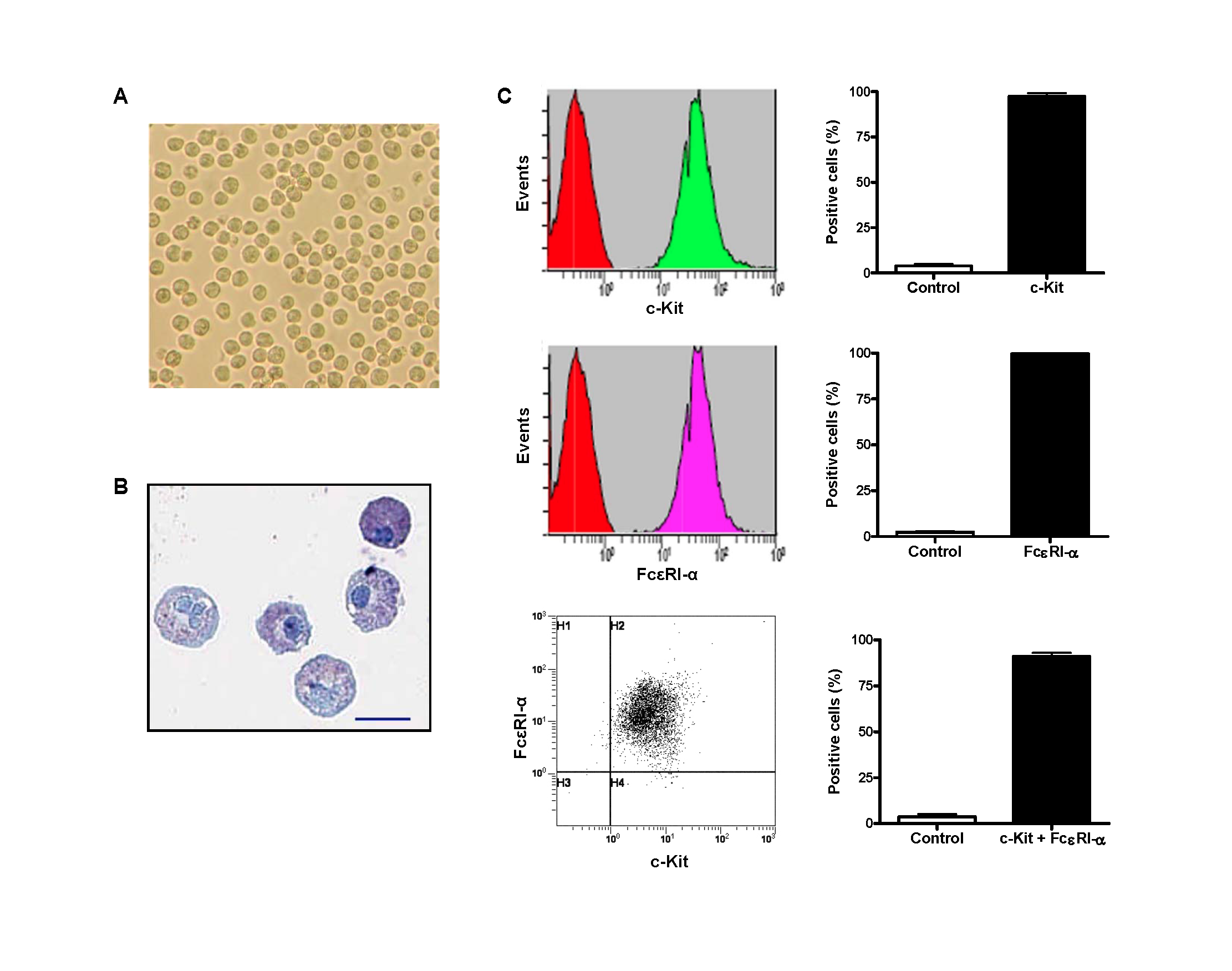

Supplement: Supplementary file 1 — Figure S1 Identification and characterization of mast cells (MCs). [file JCMM-19-2751-s001.tiff]
