# Supplementary material for: The cardiac repair benefits of inflammation do not persist: evidence from mast cell implantation
Source: J Cell Mol Med. 2015 Oct 16;19(12):2751–62. doi: 10.1111/jcmm.12703 (PMC4687709; doi:10.1111/jcmm.12703)
Supplement: Supplementary file 2 — Data S1 Supplemental methods. [file JCMM-19-2751-s002.doc]

**Supplemental Information**

**Supplemental Methods**

***Animal procedures***

All animals received humane care according to the *Guide for the Care and Use of Laboratory Animals, 8^th^ edition* (NIH, revised 2011), and all experimental procedures were approved by the Animal Care Committee of the University Health Network. Female C57BL/6 mice 8–10 weeks of age were obtained from Charles River Laboratories and c-Kit deficient mice were obtained from The Jackson Laboratory. Mice were intubated and ventilated with 2% isoflurane (Pharmaceutical Partners of Canada). Through a thoracotomy, the pericardium was dissected and the left anterior descending coronary artery was ligated. Sham mice underwent thoracotomy without ligation. Cell transplantation was performed immediately following coronary occlusion: 3×10^5^ cells were suspended in 15μL of serum-free medium and delivered in three injections across the area subtended by the ligated coronary artery in a predetermined injection protocol that was consistent among the groups. Control mice received medium with no cells.

***Preparation of cells***

Bone marrow (BM) cells were obtained by flushing the marrow cavity of the tibias and femurs of 6- to 8-week-old C57BL/6 mice. After isolation, the cells were gently separated twice with lysis buffer [0.16M NH_4_Cl, 0.017M Tris (pH 7.65)] and neutralized with IMDM (Life Technologies) containing 10% FBS (Life Technologies). The cells were then resuspended in serum-free IMDM in preparation for transplantation.

Mast cells (MCs) were obtained by flushing the marrow cavities of 6- to 8-week-old C57BL/6 mice. Cells were cultured in 5% OPTI-MEM (Life Technologies) containing 6% WEHI-3 (ATCC), which includes the IL-3 necessary for MC survival. The number of MCs was maintained at 0.5–2×10^6^/mL. After 1 month of culture, the MCs were used in experiments.

#### *Cardiac function and morphometry*

#### Cardiac function was evaluated by echocardiography. Mice were sedated with 2% isoflurane and echocardiographic images were recorded as previously described [1]. Left ventricular (LV) end-diastolic diameter (LVEDd), end-systolic diameter (LVEDs), end-diastolic area (LVEAd), and end-systolic area (LVEAs) were measured. Percent fractional shortening (%FS) and percent ejection fraction (%EF) of the LV were calculated as follows: %FS = [(LVEDd – LVEDs)/LVEDd] × 100; %EF = [(LVEDd)^2^ – (LVEDs)2/(LVEDd)^2^] × 100.

Seven and 28 days after myocardial infarction (MI), pressure–volume analysis was performed (n = 9/group). To fluid-load the animals, 1mL of heparinized saline (Baxter Canada) was injected intraperitoneally 30min before the procedure. Under positive pressure ventilation, a 1.4F micromanometer and conductance catheter (Millar Instruments) was introduced into LV through the right carotid artery. Pressure–volume loops were obtained during brief periods of apnea and before and after inferior vena cava occlusion.

After functional analysis was complete (28 days after MI), the hearts were arrested and fixed at physiologic pressure with 10% formalin (Sigma-Aldrich, n = 5/group). The hearts were cut into 1mm sections and photographed. Scar area was measured by computed planimetry using the ImageJ software (NIH) and expressed as a percentage of the LV free wall area.

***Flow cytometry analysis***

MCs were isolated from BM and labeled with PE-conjugated antibodies against c-Kit and FcεRI-α (the α-chain of the high-affinity receptor for the Fc region of immunoglobulin E, BD Biosciences). PE-conjugated IgEhb served as the isotype control.

Mouse hearts were collected at 1, 3, and 7 days post-MI and separated into infarct and non-infarct segments prior to digestion with 0.1% collagenase type II (Worthington) at 37°C for 30min. After filtering through a 70µm filter, the cells were resuspended in PBS supplemented with 2% FBS and 0.1% sodium azide (Sigma-Aldrich). 10^6^ cells were collected for staining with FITC-conjugated rat anti-mouse neutrophil (AbD Serotec) or rat anti-mouse F4/80 (Abcam) antibodies. All antibody incubations were performed for 30min at 4°C in the dark. Alexa Fluor 488-conjugated donkey anti-rat (Molecular Probes) was added as a secondary antibody when using anti-mouse F4/80. Isotype-identical antibodies served as controls (BD Biosciences). Cells were analyzed using an EPICS XL flow cytometer with EXPO32 ADC software (Beckman Coulter). The fluorescence intensity of 10,000 cells for each sample was quantified.

***Quantification of myocardial cytokine levels***

The heart samples were separated into infarcted and non-infarcted regions and homogenized in liquid nitrogen. Total protein was extracted from powdered tissue in lysis buffer [20mM Tris (pH 7.4), 150mM NaCl, 1mM EDTA, 1mM EGTA, 1% Triton X-100, 2.5mM sodium pyrophosphate (Sigma-Aldrich), 1mM β-glycerolphosphate (Sigma-Aldrich), 1mM Na_3_VO_4_ (Sigma-Aldrich), 1μg/mL leupeptin (Sigma-Aldrich), 1μg/mL pepstatin (Sigma-Aldrich), and 1mM phenylmethylsulfonyl fluoride (Sigma-Aldrich)] for 1h on ice. After centrifugation at 10,000×*g* for 10min, the supernatant was collected and protein concentration was determined using a DC protein assay kit (Bio-Rad). The levels of TNF-α (Life Technologies), TGF-β, and bFGF (R&D Systems) were determined using ELISA following the manufacturer’s instructions and expressed as pg/mg total protein.

***Collagen gel contraction assay***

Collagen gels were prepared with rat tail collagen (BD Biosciences) diluted with 0.02N acetic acid so that the final mixture contained 2.56mg/mL collagen. Cardiac fibroblasts (2×10^5^ cells) were trypsinized [0.05% trypsin (Life Technologies), 0.53mM EDTA (GIBCO)] and mixed with MCs at a 1:1 ratio in 2× IMDM. The cells were mixed with the neutralized collagen solution to a final collagen concentration of 1.28mg/mL. Aliquots (1mL/well) of the cell/collagen mixture were cast into each well of a 24-well tissue culture plate (Corning Life Sciences) and allowed to gel at room temperature. After gelation was complete (normally within 20min), the gels were gently released and transferred to 60mm tissue culture dishes (3 gels/dish) containing 5mL of freshly prepared DMEM (Life Technologies) containing 5% FBS. The gels were incubated at 37°C in a 5% CO_2_ atmosphere for up to 2 days. To explore the effects of TGF-β, gels were incubated with 5ng/mL recombinant TGF-β (R&D Systems) or a TGF-β neutralizing antibody (2.5µg/mL, R&D Systems). Gel size was measured using the AlphaImager 2200 software (ProteinSimple) and expressed as percent shrinkage compared with the blank control gel.

***MTT assay***

WT C57BL/6 or *Kit^W/W-v^* MCs were co-cultured with WT C57BL/6 fibroblasts for up to 6 days and fibroblast proliferation was measured using an MTT assay. To explore the effects of bFGF, gels were incubated with 5ng/mL recombinant bFGF (R&D Systems) or an FGF-2-neutralizing antibody (7.5µg/mL, EMD Millipore). MTT (Sigma-Aldrich) was prepared in PBS to a final concentration of 1mg/mL. The media was removed from the cells, and 50μL of MTT was added and incubated for 3–4h at 37°C. For co-culture studies, the wells were washed twice with PBS before adding the MTT. Untransformed MTT was carefully removed, and 50μL of propanol was added to each well. Optical density was measured using an automatic plate reader at 560nm with a reference wavelength of 690nm.

***Immunohistochemistry***

To determine capillary and myofibroblast density in the myocardium, the LV was perfused with 0.9% saline, embedded in OCT compound (Sakura Finetek), and then snap-frozen in liquid nitrogen. 5μm transverse sections were stained with Alexa Flour568-labeled isolectin GS-IB4 (Life Technologies) and anti-α-SMA (Sigma-Aldrich). Alexa 488-conjugated secondary antibody (Life Technologies) was used for detection of α-SMA. 4',6-diamidino-2-phenylindole (DAPI, Sigma-Aldrich) was used to stain the nuclei. The capillary density and number of myofibroblast structures were determined per high-power field with a fluorescent microscope (Nikon) by counting five randomly selected fields per section.

Using ImageJ software, the isolectin^+^ and α-SMA^+^ pixel areas were measured and expressed as the percentage of total possible pixels, after SMA^+^-coated blood vessels (≥30µm) were digitally removed.

***Statistics***

Data are presented as mean ± SD. GraphPad Prism was used for statistical analysis. Comparisons among three or more groups were performed with one- or two-way ANOVA, with differences specified by Tukey or Bonferroni post-hoc tests, respectively. A value of *P* < 0.05 was considered statistically significant.

**References**

[1**] Fazel S, Cimini M, Chen L, et a**l. Cardioprotective c-kit+ cells are from the bone marrow and regulate the myocardial balance of angiogenic cytokines*. J. Clin. Inves*t. 2006; DOI: 10.1172/JCI27019.

**Supplementary Figure 1: Identification and characterization of mast cells (MCs).**

**(A)** MCs isolated from the bone marrow of mice were cultured for 30 days (100× magnification). **(B)** MCs were stained with toluidine blue to identify granules (scale bar = 50μm). **(C)** The purity of the MCs was assessed by flow cytometry with PE-conjugated antibodies against c-Kit and FcεRI-α (n = 3/group).
